# First Case of Human Primary Vertebral Cystic Echinococcosis Due to *Echinococcus Ortleppi*

**DOI:** 10.3390/jcm7110443

**Published:** 2018-11-15

**Authors:** Louise Basmaciyan, Bénédicte Burlet, Selim Ramla, Mathieu Blot, Sophie Mahy, Marie-Hélène Aubriot-Lorton, Stéphane Valot, Mickael Grelat, Marc Sautour, Frederic Grenouillet, Jenny Knapp, Laurence Millon, Lionel Piroth, Laurent Martin, Frederic Dalle

**Affiliations:** 1 Department of Parasitology and Mycology, University Hospital, F-21000 Dijon, France; louise.basmaciyan@chu-dijon.fr (L.B.); benedicte.burlet@chu-dijon.fr (B.B.); stephane.valot@chu-dijon.fr (S.V.); marc.sautour@chu-dijon.fr (M.S.); 2 Department of Pathology, University Hospital, F-21000 Dijon, France; selim.ramla@chu-dijon.fr (S.R.); marie-helene.lorton@chu-dijon.fr (M.-H.A.-L.); laurent.martin@chu-dijon.fr (L.M.); 3 Department of Infectiology, University Hospital, F-21000 Dijon, France; mathieu.blot@chu-dijon.fr (M.B.); sophie.mahy@chu-dijon.fr (S.M.); lionel.piroth@chu-dijon.fr (L.P.); 4 Department of Neurosurgery, University Hospital, F-21000 Dijon, France; mickael.grelat@chu-dijon.fr; 5 WHO Collaborating Centre for Prevention and Treatment of Echinococcosis, and French National Reference Centre for Echinococcosis, University Hospital, F-25030 Besançon, France; fgrenouillet@chu-besancon.fr (F.G.); jknapp@chu-besancon.fr (J.K.); lmillon@chu-besancon.fr (L.M.)

**Keywords:** vertebral cystic echinococcosis, *Echinococcus ortleppi*, human

## Abstract

Cystic echinococcosis (CE) is a cosmopolitan parasitic zoonosis affecting more than one million people worldwide. In humans, primary bone CE is rare and involvement of *E. ortleppi* is very uncommon. We report here the first case of primary vertebral cystic echinococcosis due to *E. ortleppi* in Burgundy, France.

## 1. Introduction

Cystic echinococcosis (CE) is an endemic cosmopolitan parasitic zoonosis affecting approximately 200,000 incident cases a year mainly in rural areas and livestock regions [1].

The causative agent is the larval stage of the taeniid tapeworm *Echinococcus granulosus* sensu lato (s.l.). The term ‘*Echinococcus granulosus* s.l.’ includes five cryptic species: *E. granulosus* sensu stricto (s.s.), *E. equinus*, *E. ortleppi*, *E. canadensis* and *E. felidis* [2]. These species vary in terms of geographical distribution, host specificities and pathogenicity. Thus, *E. granulosus* s.s. is the most described pathogen in humans, accounting for more than 88% of the cases of human CE [3]. In humans, primary CE mainly affects the liver (i.e., 70% of cases) but can also occur in other organs, such as the lung, in 20% of cases. The bone is rarely involved, in only 0.5% to 4% of cases [2].

Usually, the transmission of human CE is accidental, resulting from consumption of water, food or soil contaminated by infected dog stools [4]. Thus, the most common predisposing factor to human CE remains the close proximity of humans to infected dogs (e.g., sheepdogs, farm dogs, free roaming dogs) [5,6]. Furthermore, other risk factors such as pastoral occupation, poor education, age, sex or drinking water source have been already described [7,8].

Recently, we published the picture of a vertebral cystic echinococcosis observed by magnetic resonance imaging (i.e., lobulated lesion of the ninth thoracic vertebra with an epidural component) in the New England Journal of Medicine [9], raising strong interest in the medical and scientific communities, in particular with regard to the pathophysiological and parasitological aspects.

We collected clinical and epidemiological complementary data, and our recent molecular investigations identified the etiological agent as an *Echinococcus* species that is very rare in human disease, *E. ortleppi.* Only eight cases have been reported worldwide since 1984. In addition, this species had never been described in humans to cause primary vertebral cystic echinococcosis, raising new epidemiological and pathophysiological issues and developments.

We report here the first case of primary vertebral cystic echinococcosis due to *E. ortleppi* in a 35-year-old woman without predisposing risk factors to bone involvement (e.g., antecedent of neoplastic syndrome or tuberculosis) in June 2017 in Burgundy, France.

## 2. Case Report

A 35-year-old woman, with no particular predisposing risk factors, consulted for a progressive motor deficit with loss of balance. Her past medical history revealed the presence of dysesthesias of the lower limbs with wet foot sensation and heat in the thighs since January 2017. These dysaesthesias progressively worsened over six months until the loss of balance and the appearance of a motor deficit leading to falls. At admission (24 May 2017), the clinical examination showed a motor deficit of the right foot elevator muscle and a sensory disturbance of the lower limbs. The biological investigations reported an inflammatory syndrome with thrombocytosis and inflammatory anemia without hyper-eosinophilia.

**Figure 1:**
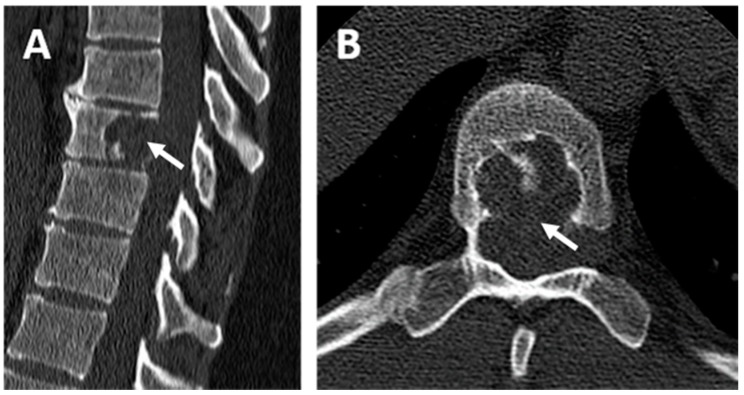
Computed tomography scan (CT scan) of the patient’s spine. (**A**) Dorsal CT scan in the sagittal plane and (**B**) dorsal CT scan in the axial plane centered on the ninth dorsal vertebra (T9). Osteolytic lesion containing septa centered on the body of the ninth dorsal vertebra (white arrow) with lysis of the posterior wall without osteo-condensation and fluid density.

The magnetic resonance imaging (MRI) and the computed tomography scan (CT scan) revealed a spinal lesion of the ninth dorsal vertebra (Figure 1). The lesion was an encapsulated multi-compartmented cyst of about 15 mm height involved in medullary compression, suggesting at first an aneurysmal bone cyst associated with shrinkage of the medullary canal and compression of the spinal cord. A posterior corporectomy of the ninth dorsal vertebra (T9) with laminectomy and osteosynthesis were performed, associated with a complete exeresis of the lesion, which was composed of fibrous tissue consisting of several rounded cavities developed from the ninth dorsal vertebra, causing spinal cord compression. Multiples biopsies of the lesion were performed for microbiological and histopathological investigations.

**Figure 2:**
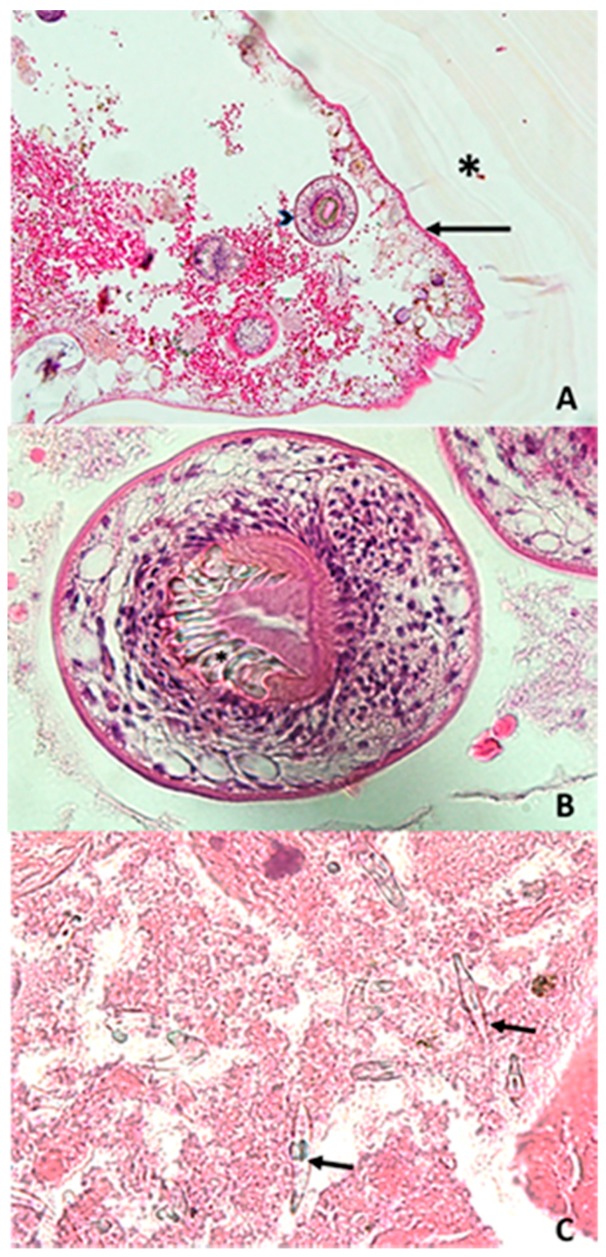
Microscopic observation of the samples collected from the ninth dorsal vertebra after hematoxylin and eosin staining. (**A**) Laminated layer (*) in contact with the proliferating membrane (black arrow), from which protoscoleces (arrow head) are detached (200×); (**B**) Protoscoleces in the cyst lumen with visible hooks (*) (600×); (**C**) Free hooks (black arrow) (1000×).

Histopathological and parasitological analyses reported fragments of the typical laminated layered structure of a hydatid cyst (Figure 2A). On the internal surface of the hydatid cyst, the germinative membrane displayed cuboid cells with abundant eosinophilic cytoplasm (Figure 2A). Inside the cystic structure, protoscoleces were observed with visible hooks (Figure 2B), and free hooks were also observed (Figure 2C). Serological investigations were carried out by the French National Reference Center for Echinococcosis (FNRCE), which reported the presence of anti-*Echinococcus* antibodies (i.e., positive *E. granulosus* hemagglutination (Fumouze, Levallois, France) at a 1:640 titer and a positive Western blot with a p7 and p26/28 positive band pattern (LDBio Products, Lyon, France)), although these data were not able to differentiate a cystic echinococcosis from an alveolar echinococcosis [10]. DNA extraction was carried out from a paraffin-embedded T9 biopsy fragment using the QIAamp DNA mini kit (Qiagen, Hilden, Germany) and then sent to the FNRCE for molecular identification of the *Echinococcus* species involved in the cystic lesion. Specific PCRs targeting the *E. multilocularis* [11] and *E. granulosus* s.s. [12] 12S RNA mitochondrial gene (target sizes of 200 bp and 255 bp, respectively) were negative, while a specific *Echinococcus* spp. PCR targeting a 350 bp fragment [13] of the 12S RNA mitochondrial gene with primers (12S-Echino-Fwd: 5’-AAAKGGTTTGGCAGTGAGYGA-3’; 12S-Echino-Rev: 5’-GCGGTGTGTACCTGAGCTAAAC-3’) designed to amplify all *Echinococcus* species was positive. DNA sequencing allowed the identification of *Echinococcus ortleppi* by comparison with the online genetic databases, by using the Basic Local Alignment Search Tool (BLAST) available on the NCBI website. A 100% identity was obtained with the reference sequence KY766908.1 on a 201 bp sequence alignment [14].

After surgery, oral albendazole 800 mg/day was initiated for a planned total duration of two years. The thoraco-abdomino-pelvic CT scan reported no arguments for hepatic, pulmonary or visceral echinococcosis. No infectious intercurrent event was observed after surgery and anti-parasitical therapy. Six months later, the clinical evolution was favorable with normal neurological examination. Serologic investigations carried out at the FNRCE reported a significant decrease in hemagglutination antibody titers and a lower intensity profile in Western blot.

## 3. Discussion and Conclusions

According to World Health Organization (WHO) reports in 2017, infection by *E. granulosus* s.l. has led to approximatively 200,000 CE incident cases per year. In France, analysis of nationwide hospital information databases revealed an average annual incidence rate of CE of about 0.42 cases per 100,000 inhabitants between 2005 and 2014 [15].

Our patient presented with a primary vertebral cyst at the ninth dorsal vertebra with symptomatic medullar compression. Half of the cases of vertebral cystic echinococcosis occur at the dorsal spine, followed by the lumbar spine (37%) and rarely at the sacral and cervical spine (5.5% and 5.5%, respectively) [16]. Thus, a case with a primary dorsal vertebral cyst is rare, with less than 0.5% of primary CE occurring at this location.

*Echinococcus granulosus* s.l. is the causative agent of CE in animals and humans. Nowadays, mitochondrial DNA sequencing has differentiated 10 genotypes (G1 to G10). A recent taxonomical revision has grouped genotypes G1 to G3 under the name *E. granulosus* s.s. and the genotypic cluster G6 to G10 under the name *E. canadensis*. Additionally, the specific name *E. equinus* was attributed to G4 and *E. ortleppi* to G5. Finally the ‘lion strain’ was named *E. felidis* [2]. These species vary in terms of geographical distribution, host specificities and pathogenicity. Thus, *E. granulosus* s.s. is the most described pathogen in humans, with more than 88% of human CE attributed to it [3]. Moreover, among *E. granulosus* s.s., the genotype G1 has the most cosmopolitan distribution, often associated with the transmission by sheep (an intermediate host) [3].

First described in South Africa, *E. ortleppi* (genotype G5) has a dog/cattle life cycle and a sporadic worldwide distribution [2,17]. While classically reported in animals in many countries, *E. ortleppi* is very uncommon in humans, with only eight cases reported worldwide since 1984 (Table 1) and 75% of these lesions being localized in the liver. In France, the two human cases reported occurred in two different regions (Jura and Vendée) and do not match the French cattle foci of CE [17], raising the question of unexplored zoonotic foci of *E. ortleppi* transmission in France.

Nevertheless, the importance of *E. ortleppi* remains largely unknown, which highlights the need for enhanced survey efforts. Indeed, current serological diagnostic tools are based on the use of antigens from *E. granulosus* s.s. G1, which may differ from *E. ortleppi* antigens [17]. Moreover, gene amplification and sequencing approaches are not available in routine microbiology laboratories worldwide. Thus, human cases of cystic echinococcosis due to *E. ortleppi* are probably underestimated, contributing to the poor knowledge available regarding the pathogenicity of this species in humans [17].

## Figures and Tables

**Table 1:** Cases of cystic echinococcosis involving *E. ortleppi* since 1984.

Years	Age (Years)	Sex	Country (Region)	Clinical Statement	Cyst Localization	References
2011	63	M	Eastern France (Jura)	Moderate pain in right hypochondrium	Liver	[17]
2012	39	F	Western France (Vendée)	Abdominal pain, fever	Liver	[17]
2010–2012	*	*	South Africa (Ganteng Province)	*	Liver	[18]
2011–2012	*	M	North India (Uttarakhand)	*	Liver	[19]
2002	*	*	Argentina	*	Liver	[20]
2004	38	F	Central Mexico	Intense pain in right hypochondrium	Liver	[21]
1984	11	M	Netherlands	*	Spleen	[22]
*	*	*	Brazil (Santana do Livramento)	*	*	[23]

* Unspecified.

## Abbreviations

CE	Cystic Echinococcosis
CT scan	Computed Tomography Scan
MRI	Magnetic Resonance Imaging
s.l.	Sensu Lato
s.s.	Sensu Stricto
